# Distinct microbiota assembly mechanisms revealed in different reconstruction stages during gut regeneration in the sea cucumber *Apostichopus japonicus*

**DOI:** 10.1002/mbo3.1250

**Published:** 2021-11-22

**Authors:** Zichao Yu, Zhuang Xue, Chao Liu, Anguo Zhang, Qiang Fu, Kun Yang, Fang Zhang, Liyuan Ran

**Affiliations:** ^1^ School of Laboratory Animal & Shandong Laboratory Animal Center Shandong First Medical University & Shandong Academy of Medical Sciences Jinan China; ^2^ Liaoning Key Laboratory of Marine Animal Immunology Dalian Ocean University Dalian China; ^3^ National Marine Environmental Monitoring Center, Ministry of Ecology and Environment Dalian China

**Keywords:** *Apostichopus japonicus*, evisceration, gut microbiota reconstruction, gut regeneration

## Abstract

*Apostichopus japonicus* is a useful model for studying organ regeneration, and the gut microbiota is important for host organ regeneration. However, the reconstruction process and the mechanisms of gut microbiota assembly during gut regeneration in sea cucumbers have not been well studied. In the present study, gut regeneration was induced (via evisceration) in *A*. *japonicus*, and gut immune responses and bacterial diversity were investigated to reveal gut microbiota assembly and its possible mechanisms during gut regeneration. The results revealed that bacterial community reconstruction involved two stages with distinct assembly mechanisms, where the reconstructed community was initiated from the bacterial consortium in the residual digestive tract and tended to form a novel microbiota in the later stage of reconstruction. Together, the results of immunoenzyme assays, community phylogenetic analysis, and source tracking suggested that the host deterministic process was stronger in the initial stage than in the later stage. The bacterial interactions that occurred were significantly different between the two stages. Positive interactions dominated in the initial stage, while more complex and competitive interactions developed in the later stage. Such a dynamic bacterial community could provide the host with energetic and immune benefits that promote gut regeneration and functional recovery. The results of the present study provide insights into the processes and mechanisms of gut microbiota assembly during intestinal regeneration that are valuable for understanding gut regeneration mechanisms mediated by the microbiota.

## Graphical abstract

Ejection of internal organs was induced in the sea cucumber *Apostichopus japonicus*, and gut immune responses and bacterial diversity during gut regeneration were investigated. We revealed that the reconstruction of the bacterial community involved two stages. The diversity, taxonomic composition, and bacterial interaction of the gut bacterial community and the interaction between the bacterial community and the host were significantly different in the two stages.

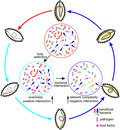

## 1 INTRODUCTION

The sea cucumber *Apostichopus japonicus* possesses a unique defense mechanism called evisceration, a process in which it ejects all of its internal organs upon encountering predators or environmental stress and then regenerates the internal organs, which recover their normal functions within a few weeks (Byrne, 1985; Dolmatov & Ginanova, 2009; Shukalyuk & Dolmatov, 2001). Evisceration can be easily induced with KCl in the laboratory (Byrne, 1985), and using a consistent and repeatable method for the induction of evisceration can minimize the variation among individuals with regard to the extent and severity of the trauma. Moreover, gut regeneration decouples host ontogeny from gut development (Weigel, 2020). These factors make sea cucumbers a useful model for studying organ regeneration and development (Mashanov & García‐Arrarás, 2011; Mashanov et al., 2010).

The commensal microbiota plays a crucial role in governing the health and behavior of its host (McFall‐Ngai et al., 2013; Thaiss et al., 2016; Wang & Jia, 2016) by preventing damage from pathogens (Defer et al., 2013), modulating the host immune system (Schmitt et al., 2012), and promoting nutrient absorption by the host (Yamazaki et al., 2016). The endogenous microbiota is important for organ regeneration and development in both vertebrates and invertebrates, participating in processes such as hematopoiesis in mice (Josefsdottir et al., 2017), gut differentiation in zebrafish (Bates et al., 2006; Cheesman et al., 2010), and tissue regeneration in planaria (Arnold et al., 2016). In sea cucumber, gut regeneration is accompanied by the reconstruction of the gut microbiota, and the results of a recent study indicated that the rate of gut regeneration in sea cucumber is associated with its gut microbiota (Zhang et al., 2020). Understanding the ecological mechanisms underlying the microbial community dynamics that occur during host development is a current research priority and valuable for better understanding the mechanisms underlying host development and guiding the manipulation of the gut microbiota to improve host health, especially in aquatic animals (De Schryver & Vadstein, 2014; Pérez‐Sánchez et al., 2018). Gut regeneration in sea cucumber provides a useful model for investigating the developmental process and associated mechanisms of the gut microbiota of aquatic invertebrates. The gut microbial community is considered to be affected by internal factors (i.e., host selection and interactions among microbial populations) and external (i.e., environmental) factors (Bakke et al., 2013; De Schryver & Vadstein, 2014). Investigating the factors governing host gut microbiota assemblage and microbe‐microbe interactions during gut regeneration in the sea cucumber will provide a better understanding of the ecological mechanisms involved, and the mechanisms associated with marine animal organ development mediated by the commensal microbiota. In the past few years, an increasing number of studies on the gut microbiota of sea cucumbers have focused on its bacterial composition, physiological characteristics, and potential effects on host health (Gao et al., 2014; Pagán‐Jiménez et al., 2019; Sha et al., 2016; Yamazaki et al., 2016). Although the variations in gut bacterial community composition and functional genes during gut regeneration in *A*. *japonicus* have been studied by two research groups (Wang et al., 2018; Zhang et al., 2019), the dynamics of the gut microbial community and the interactions that occur in the microecosystem during *A*. *japonicus* gut regeneration remain poorly understood.

In the present study, immunoenzyme activities during different regeneration stages of the *A*. *japonicus* gut were measured, and the diversity of the bacterial communities in the gut and rearing seawater was investigated through 16S rRNA gene sequencing. The goals of the present study were to (1) describe bacterial community succession during *A*. *japonicus* gut regeneration, (2) identify the primary factors governing *A*. *japonicus* gut bacterial community assembly in different reconstruction stages, and (3) elucidate the ecological mechanisms underlying bacterial dynamics during *A*. *japonicus* gut regeneration.

## 2 MATERIALS AND METHODS

### 2.1 Experimental design

**FIGURE A1:**
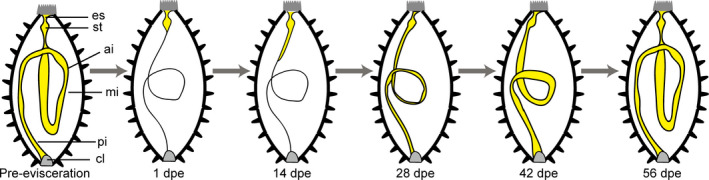
Gut regeneration process of *A*. *japonicus*. es, esophagus, st, stomach, ai, anterior intestine, mi, middle intestine, pi, posterior intestine, vm, ventral mesentery, dm, dorsal mesentery, cl, cloaca; yellow‐colored area, the tissue collected for microbiota analysis

Sea cucumbers (*A*. *japonicus*) were collected from a local aquatic farm in Dalian, Liaoning Province, China, and were acclimated in aerated seawater at 10–12°C. All the sea cucumbers were kept in one tank and fed every 2 days with a sterile commercial diet (Haijie), and two‐thirds of the water was changed before feeding. The sea cucumbers were induced to eviscerate by the injection of 2 ml of sterile 0.35 M KCl into the coelom and then cultured in aerated seawater at 10–12°C to allow regeneration of the viscera. Evisceration results in the loss of the digestive tract between the stomach and cloaca (Wang & Li, 2007). The regeneration process is mainly divided into the stages of wound healing (0–1 dpe), intestine lumen forming (1–28 dpe), differentiation, and growth stage (28–56 dpe; Figure A1). Sea cucumbers were randomly sampled before evisceration (Pre group), and at different stages during the regeneration. Fifteen sea cucumbers were sampled at each of 0 and 1 dpe, and nine individuals were sampled at each of the other sampling times. The tissues were sampled after the coelom was washed with sterile seawater to remove the residual coelomic fluid. For the Pre group, the esophagus, stomach, and intestine were sampled; the residual esophagus and stomach were sampled at 0 and 1 dpe; the esophagus, stomach, and regenerated intestine were sampled at 14, 28, 42, and 56 dpe (Figure A1). The collected tissues were named as gut samples in this study. The samples from five (0 and 1 dpe) or three (other sampling times) individuals at each sampling time were pooled together such that three replicate samples were obtained for each sampling time for immune parameter assays and 16S rRNA gene sequencing. Seawater samples were also collected with the gut samples at 0, 14, 28, 42, and 56 dpe. Each seawater sample (1 L) was filtered through a 0.22 µm pore size membrane (Sangon Biotech) to concentrate the microbial cells. The membrane was then stored at −80°C for subsequent DNA extraction.

### 2.2 Immune parameter assays

The gut samples were homogenized in PBS buffer using a motor‐driven tissue grinder (Sangon Biotech) and centrifuged at 10,000 *g* at 4°C for 20 min, after which the supernatant was collected to measure the protein concentration and activities of immunoenzymes.

Protein concentration was determined through a Coomassie brilliant blue G assay (Löffler & Kunze, 1989) with the Total Protein Quantitative Assay Kit (Jiancheng Bioengineering Institute), and the absorbance at 595 nm was measured with a Tecan M1000 PRO plate reader (Tecan Group).

Acid and alkaline phosphatase (ACP and AKP) activities were determined based on a method described previously (Barrett & Heath, 1977) with an ACP assay kit and an AKP assay kit (Jiancheng Bioengineering Institute), respectively. One unit (U) of ACP or AKP activity was defined as the amount of phenol (mg) produced per gram protein.

Superoxide dismutase (SOD) activity was measured according to a previously described method (Ji et al., 1991) using a SOD assay kit (Jiancheng Bioengineering Institute). One U of SOD activity was defined as the amount of SOD required to inhibit 50% of superoxide‐induced oxidation, as determined by measuring the change in absorbance at 550 nm.

Lysozyme (LZM) activity was determined through a turbidity assay (Callahan et al., 2016) using an LZM assay kit (Jiancheng Bioengineering Institute). One U of LZM activity was defined as the amount of LZM that decreased the absorbance value at 530 nm by 0.001 per minute.

### 2.3 Genomic DNA extraction and sequencing

A soil DNA kit (Omega) was used to extract genomic DNA from the gut samples (three samples from each sampling time). Genomic DNA from the seawater samples (one sample from each sampling time) was extracted with a water DNA kit (Omega) following the manufacturer's instructions. DNA quality and quantity were assessed via 1% agarose gel electrophoresis and a NanoDrop spectrophotometer (Thermo Fisher Scientific, Inc). Paired‐end sequencing of the V4 region of the 16S rRNA gene in all gut and seawater samples was performed by Novogene Co., Ltd., on the Illumina MiSeq PE250 platform. The raw sequence data reported in this study have been deposited in the NCBI SRA database under accession number PRJNA720500.

### 2.4 Processing of raw DNA sequence data

The raw sequencing data were processed in QIIME2 (Bolyen et al., 2018) to analyze the diversity and taxonomic composition of the bacterial communities. Briefly, the raw FASTQ files were imported into QIIME2 and subjected to demultiplexing and quality examination. Using the DADA2 (Callahan et al., 2016) plugin, the paired‐end reads were denoised and stitched, and an amplicon sequence variant (ASV) table was generated along with the representative sequences. In the diversity analysis, the sequence counts of each sample in the ASV table were subsampled to the minimum count of all the samples to avoid bias caused by different sequencing depths. The taxonomy of each representative sequence of ASV was annotated using the Sklearn classifier algorithm against the Silva database version 132 (99% OTU dataset). Alpha diversity metrics, including the Chao1 and Pielou evenness indices, were calculated with the diversity plugin in QIIME2. To estimate the ecological processes, the standardized effect size of the mean nearest taxon distance (ses.MNTD) within single samples was computed with the picante package of R (Kembel et al., 2010), and the partitioning of the beta diversity was conducted using the betapart package in R version 3.5.3 (Baselga & Orme, 2012). The taxonomic composition of the samples was determined based on the ASV table and the taxa of the representative sequences.

### 2.5 Construction of the molecular ecological network

The network was constructed based on the abundance of each ASV. ASVs that were present in two‐thirds of the samples in each category were used for the network analyses. The Molecular Ecological Network Analysis Pipeline (MENAP, http://ieg4.rccc.ou.edu/mena/) was used to perform the network analysis based on Pearson correlation in time‐series, and the network properties were determined with an automatic threshold, as described previously (Deng et al., 2012; Zhou et al., 2010). The network was visualized in Cytoscape 3.7.1 (Shannon et al., 2003). To characterize the topology of the resulting network, a set of measures, including the average degree (avgK), average path distance (GD), R^2^ of the power law, and modularity, were calculated with MENAP. For the modularity analysis, each network was separated into several modules by fast greedy modularity optimization, and the among‐module connectivity (*Pi*) and within‐module connectivity (*Zi*) values, which indicate the connectivity of each node in the network, were calculated. The nodes were categorized into four categories as described previously (Montoya et al., 2006; Zhou et al., 2010): peripherals (*Zi* ≤ 2.5, *Pi* ≤ 0.62), connectors (*Zi* ≤ 2.5, *Pi* > 0.62), module hubs (*Zi* > 2.5, *Pi* ≤ 0.62) and network hubs (*Zi* > 2.5, *Pi* > 0.62).

### 2.6 Source tracker analysis

Source tracker analysis based on the Bayesian approach (Knights et al., 2011) was used to identify the different dispersal sources and estimate their proportional contributions to the bacterial community composition in the gut samples. Independent analyses were performed for the gut samples at each time point (identified as “sinks” in SourceTracker software). The corresponding water sample for each gut sample, the gut samples at 0 dpe, and the gut samples that preceded the “sink” samples were identified as the “sources”.

### 2.7 Statistical analyses

Statistical Package for Social Sciences (SPSS) 19.0 (SPSS Inc) was used for statistical analysis. Significant differences among groups were evaluated with analysis of variance followed by Tukey's post hoc test. The statistical significance threshold was set at *p* < 0.05, and the extreme statistical significance threshold was set at *p* < 0.01.

Principal coordinate analysis (PCoA) and constrained principal coordinate analysis (CPCoA) that was constrained by sampling time were performed in R to evaluate the differences in bacterial community structure among groups based on Bray‐Curtis distance. Similarity analysis (ANOSIM) was performed to test for significant differences among the groups. The correlations between bacterial community parameters and immune parameters at different sampling times were analyzed using CANOCO version 5.0 (Ter Braak & Šmilauer, 2012). Detrended correspondence analysis (DCA) was first performed on the taxonomic data to select an ordination method, and linear model‐based redundancy analysis (RDA) was chosen since the longest gradient was shorter than 3.0 (Lepš & Šmilauer, 2003).

## 3 RESULTS

### 3.1 Variation in bacterial community diversity during gut regeneration

**FIGURE A2:**
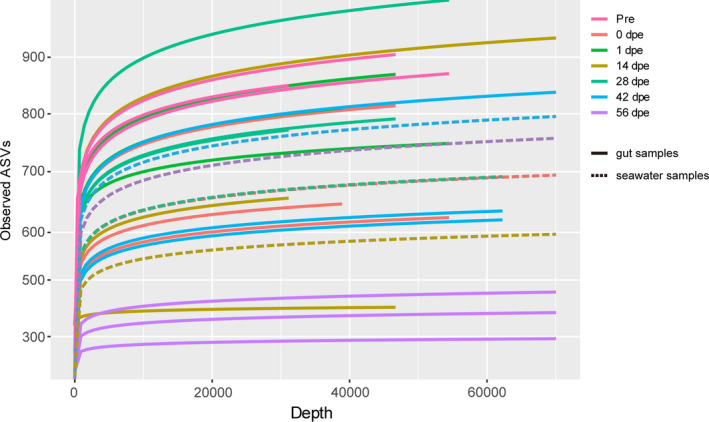
Rarefaction curves of different samples

In the present study, samples were collected before evisceration and at 0, 1, 14, 28, 42, and 56 dpe, during gut regeneration (Figure A1). A total of 1,479,755 high‐quality sequences were obtained from the gut and seawater samples, with 32,931–80,189 sequences per sample. The rarefaction curve of each sample was almost saturated (Figure A2), indicating that the sequencing depth was adequate.

**FIGURE 1:**
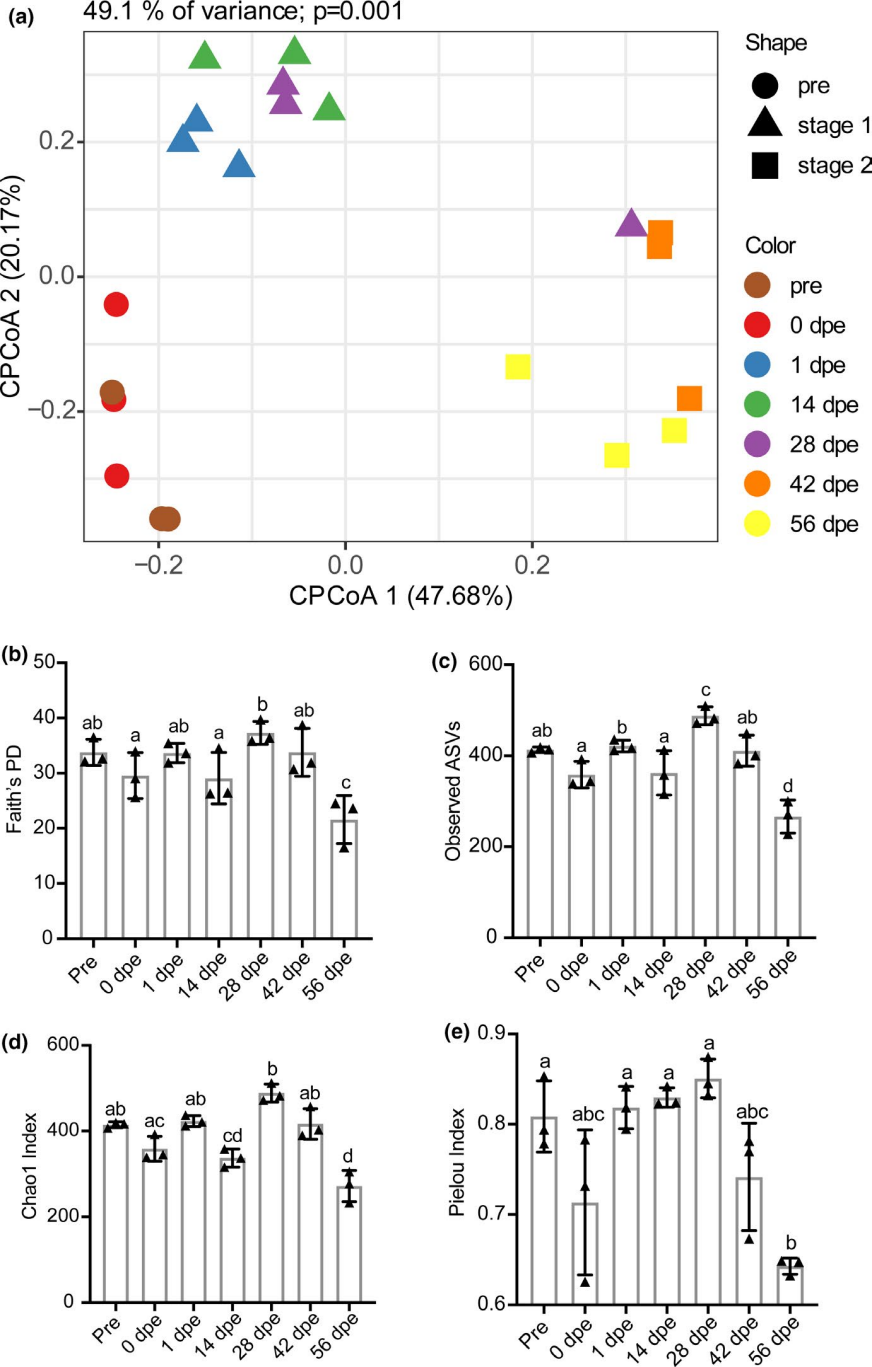
Diversity of the *A*. *japonicus* gut bacterial community at different sampling time points. (a) CPCoA analysis of the bacterial communities in the regenerating guts. (b) Variation in Faith's PD index during the sampling period. (c) Variation in the observed ASV index during the sampling period. (d) Variation in the Chao1 index during the sampling period. (e) Variation in the Pielou index during the sampling period. Different lowercase letters above the error bars indicate significant differences among groups

**FIGURE A3:**
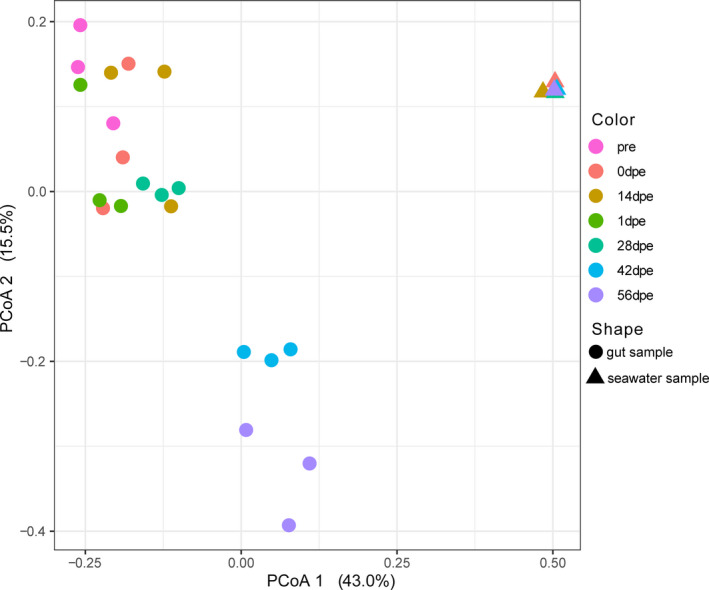
Principal coordinate analysis plot illustrating the variation of the bacterial communities in gut and seawater samples during gut regeneration

The PCoA plot (Figure A3) revealed that the gut bacterial community varied significantly during the regeneration, while the rearing seawater samples were clustered closely, indicating the bacterial communities in the rearing seawater were relatively stable during the experiment. To assess the effects of sampling time on the assembly of bacterial communities, the beta diversity of the bacterial communities was determined by CPCoA analysis (Figure 1‐a). Gut regeneration explained as much as 49.1% of the overall variance in the data. Clear clustering of the bacterial communities was observed among the gut samples, and this clustering was corroborated by ANOSIM, which yielded a global R‐value of 0.799 (*p* = 0.0001). The gut samples were grouped into three distinct clusters. Cluster 1 contained the samples collected before evisceration and at 0 dpe (representing the residual digestive tract); cluster 2 contained the regenerating gut samples collected at 1, 14, and 28 dpe; and cluster 3 contained the samples collected at 42 and 56 dpe. This pattern was confirmed by ANOSIM, in which the *p*‐value between each cluster and any other was less than 0.05 (Table A1). Based on the beta diversity analysis, we divided the gut bacterial community reconstruction into two stages, stage 1 (initial stage, 1–28 dpe) and stage 2 (later stage, 42–56 dpe).

The alpha diversity of the bacterial community was assessed with the phylogenetic diversity (Figure 1‐b), richness (Figure 1‐c,d), and evenness indices (Figure 1‐e). The Faith's PD, observed ASV and Chao1 indices exhibited similar trends, fluctuating from 1 to 28 dpe, peaking at 28 dpe, and decreasing from 42 to 56 dpe, with the lowest values observed at 56 dpe. The Pielou evenness index value increased slightly from 1 to 28 dpe and decreased from 42 to 56 dpe, with a minimum value observed at 56 dpe.

### 3.2 Composition and dynamics of the bacterial community during gut regeneration

**FIGURE 2:**
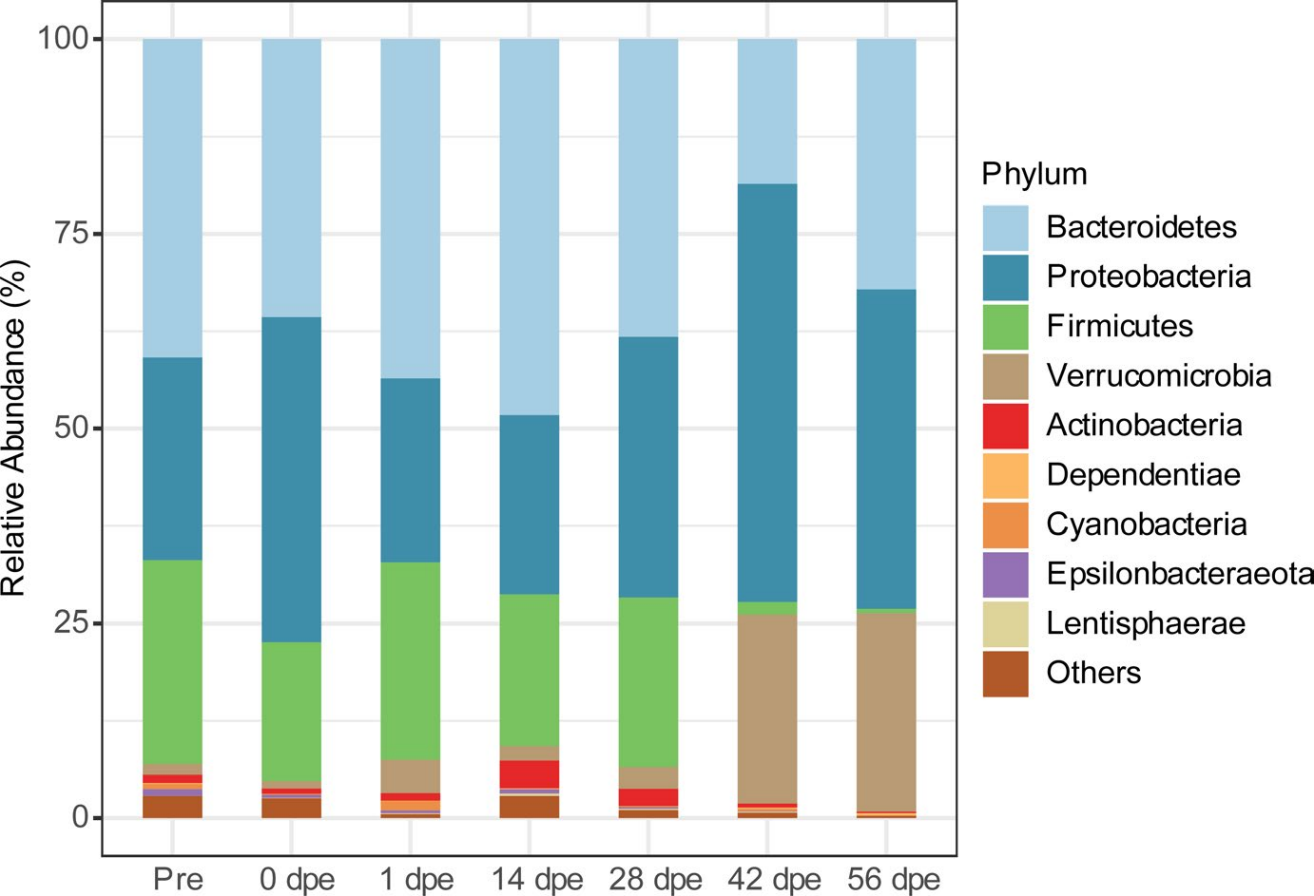
Bacterial community composition at the phylum level in different *A*. *japonicus* gut samples during gut regeneration

The gut bacterial community was dominated by the phyla *Bacteroidetes*, *Proteobacteria*, *Firmicutes*, and *Verrucomicrobia* at each sampling time of gut regeneration (Figure 2). Before evisceration, the phylum with the highest relative abundance was *Bacteroidetes* (40.90%), followed by *Firmicutes* (26.23%), *Proteobacteria* (25.97%), and *Verrucomicrobia* (1.38%). The relative abundance of *Bacteroidetes* was 35.68% in the residual digestive tract (0 dpe); it increased to 48.28% at 14 dpe, decreased to 18.59% at 42 dpe, and increased again at 56 dpe (32.11%). The relative abundance of *Proteobacteria* was 41.72% in the residual digestive tract (0 dpe). It decreased to 23.61% at 1 dpe, increased to 53.58% at 42 dpe, and then decreased at 56 dpe (41.05%). The relative abundances of *Firmicutes* and *Verrucomicrobia* displayed the opposite trend patterns. *Firmicutes* showed high abundance from 0 to 28 dpe but significantly decreased abundance at 42 and 56 dpe. The relative abundance of *Verrucomicrobia* remained low from 0 to 28 dpe but was significantly increased at 42 and 56 dpe.

**FIGURE 3:**
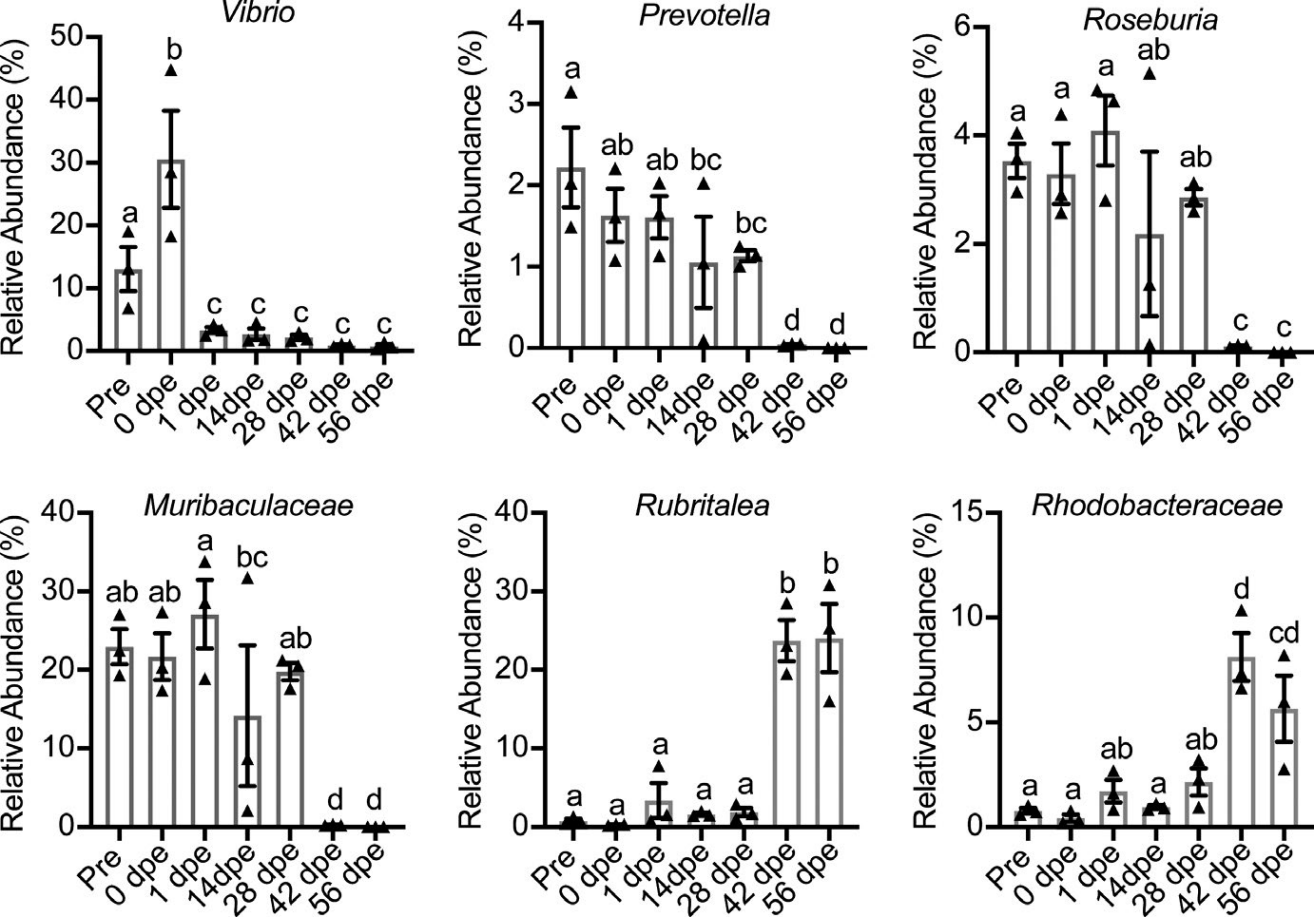
The abundances of bacterial genera at different sampling times. Different lowercase letters above the error bars indicate significant differences among groups

**FIGURE A4:**
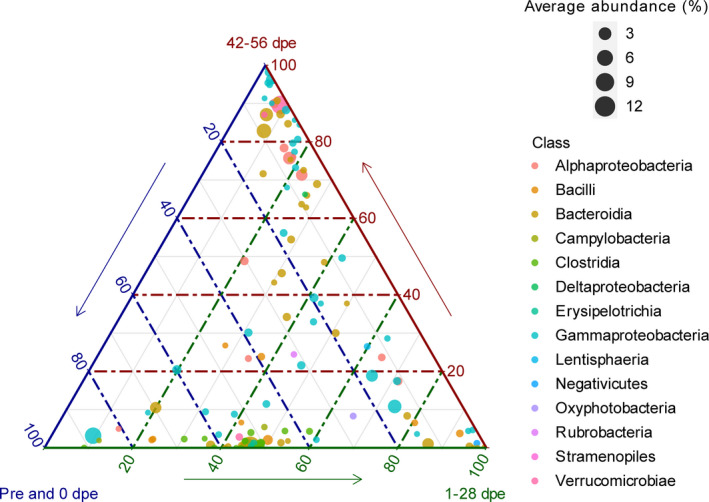
Ternary plot illustrating the succession and relative abundances of bacterial species. The size of the circle represents the relative abundance of each species, and the color represents species affiliated with different major classes

In each of the three clusters identified in the beta diversity analysis of the bacterial communities in the regenerating gut, the samples were pooled together for analysis. The succession of the dominant bacterial species in the gut regeneration process was visualized with a ternary plot (Figure A4). Bacterial taxa belonging to the classes *Gammaproteobacteria*, *Clostridia*, and *Bacteroidia* were prevalent in the samples collected before evisceration and at 0–28 dpe, while those belonging to *Gammaproteobacteria*, *Alphaproteobacteria*, *Bacteroidia*, and *Verrucomicrobiae* were abundant and ubiquitous in the gut at 42–56 dpe. For example, the abundance of *Vibrio*, a member of *Gammaproteobacteria*, was as high as 13.03% ± 6.1% and 30.54% ± 13.38% before evisceration and at 0 dpe, respectively (Figure 3). However, the abundance of this genus significantly decreased to 3.34% ± 0.85% at 1 dpe and remained significantly lower than its initial value throughout the gut regeneration process. *Prevotella* (class *Bacteroidia*), *Muribaculaceae* (class *Bacteroidia*) and *Roseburia* (class *Clostridia*) displayed the same pattern: high abundance before evisceration, at 0 dpe and from 1 to 28 dpe and significantly decreased abundance at 42 and 56 dpe. The abundances of *Rubritalea* (class *Verrucomicrobiae*) and *Rhodobacteraceae* (class *Alphaproteobacteria*) were low before evisceration and in the early regeneration period (1–28 dpe) but were increased at 42 and 56 dpe.

### 3.3 Changes in immune parameters during gut regeneration and the correlations of immune parameters with bacterial community structure

**FIGURE 4:**
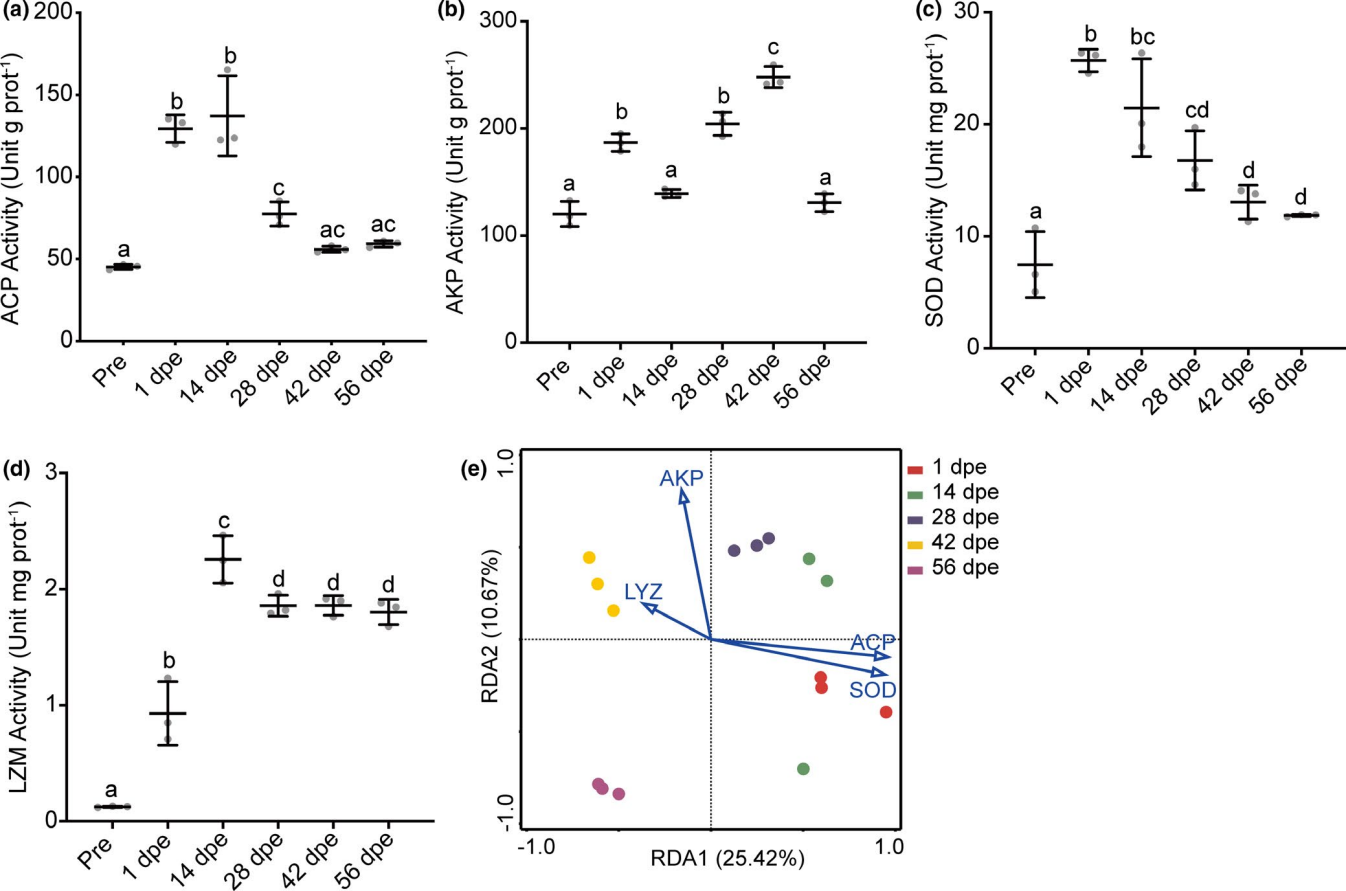
Changes in gut immunoenzyme activities (a‐d) and correlations of enzyme activities with bacterial community structure during *A*. *japonicus* gut regeneration (e). Different lowercase letters above the error bars indicate significant differences among groups

The gut activities of ACP, AKP, SOD, and LZM during gut regeneration were assessed. The activities of ACP, SOD, and LZM exhibited similar trends (Figure 4‐a,c,d), significantly increasing from 1 to 14 dpe and then decreasing until 56 dpe. AKP activity was significantly higher (*p* < 0.01) at 1 dpe than before evisceration but then decreased, showing a lower value at 14 dpe. Subsequently, AKP activity gradually increased to its maximum level at 42 dpe before significantly decreasing to the pre‐evisceration level at 56 dpe (Figure 4‐b). An RDA was performed to reveal the correlations between immune responses and the structure of the bacterial community during gut regeneration. The results indicated that ACP and SOD could be the major factors affecting bacterial community structure, with the activities of these enzymes explaining 23.9% and 23.4% of the variance in community structure (Table A2). The RDA plot also showed that the bacterial communities at 1–28 dpe were more strongly influenced by ACP and SOD than those at 42–56 dpe (Figure 4‐e).

### 3.4 Network analyses of bacterial communities during gut regeneration

**FIGURE 5:**
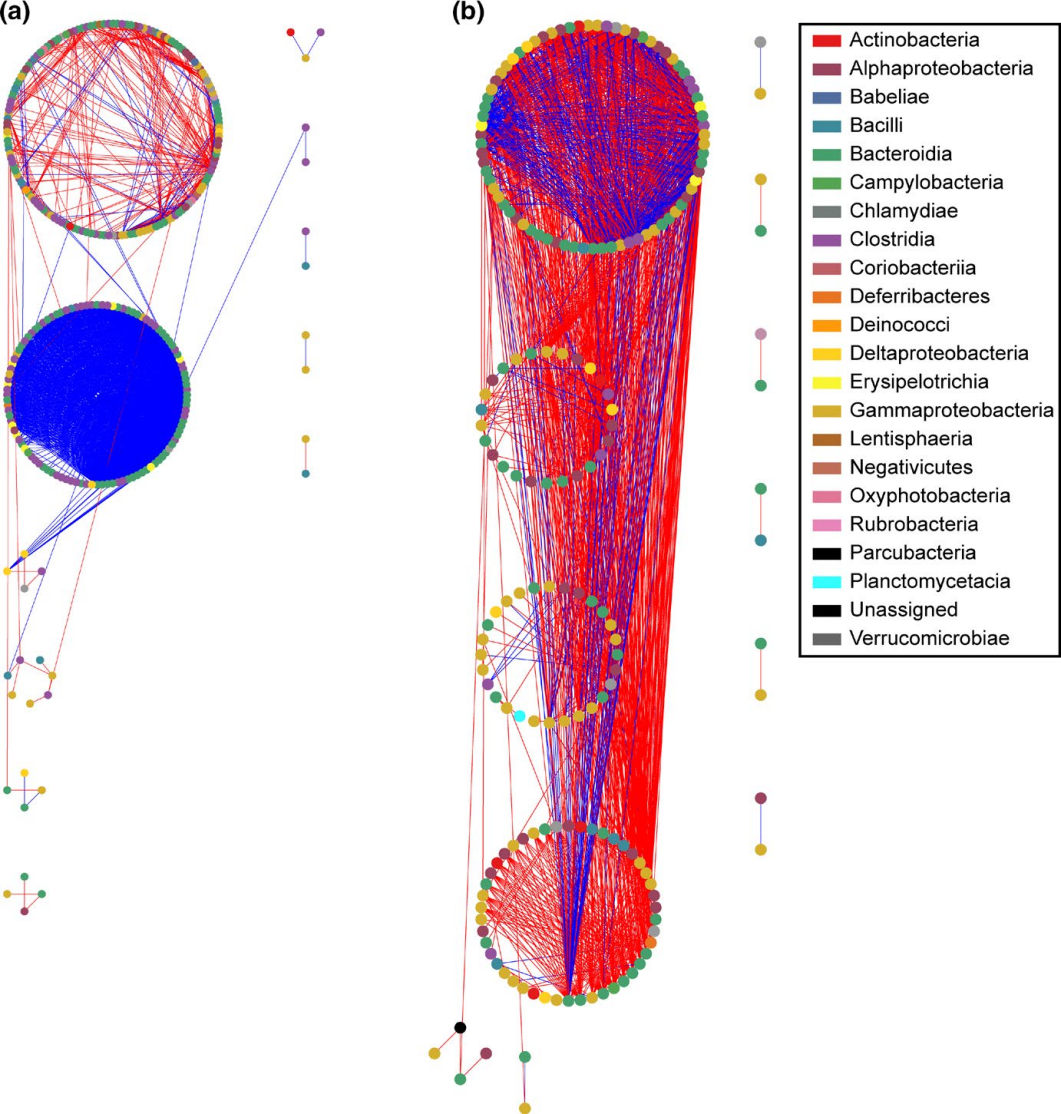
Ecological networks of the bacterial communities at different stages of *A*. *japonicus* gut regeneration. (a) Network interaction graph for the bacterial communities from 1 to 28 dpe. (b) Network interaction graph for the bacterial communities from 28 to 56 dpe. Each node represents a bacterial genus. The color of each node represents the class of the genus associated with the node. A blue edge indicates a positive interaction between two nodes, whereas a red edge indicates a negative interaction

**FIGURE A5:**
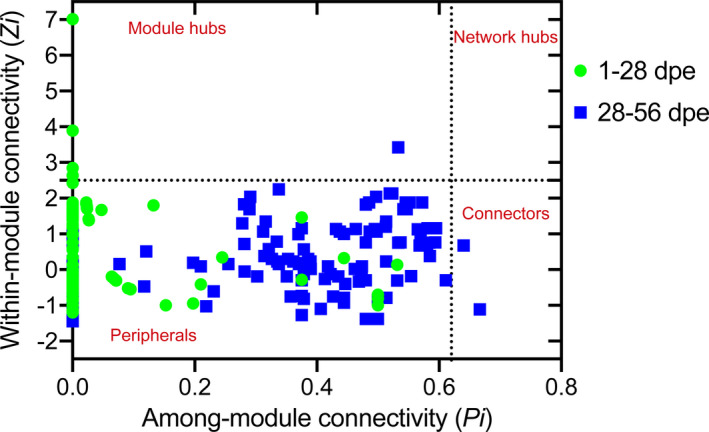
*Z*‐*P* plot illustrating the distribution of OTUs based on their topological roles. The horizontal dashed line represents *Zi* = 2.5, and the vertical dashed line represents *Pi* = 0.62

The bacterial community interactions during the different periods of gut regeneration were investigated through network analysis based on the time‐series Pearson correlation, and the topological properties of the networks were calculated (Table A3). The avgK value was highest (16.87) in the 28–56 dpe network, indicating that this network was the most complex. In addition, the percentage of positive interactions in the 1–28 dpe network was much higher (88.09%) than that in the 28–56 dpe network (28.19%; Table A3, Figure 5). The topological roles of each node in the microbial networks were determined through a Zi‐Pi graph (Figure A5). In the 1–28 dpe network, six nodes affiliated with *Clostridia*, *Alphaproteobacteria*, *Gammaproteobacteria* were classified as module hubs, and two nodes affiliated with *Clostridia* and *Bacteroidia* were classified as connectors. Two nodes that were affiliated with *Alphaproteobacteria* and *Gammaproteobacteria* functioned as connectors in the 28–56 dpe network.

### 3.5 Source tracking of the bacterial community in the regenerated gut

**FIGURE 6:**
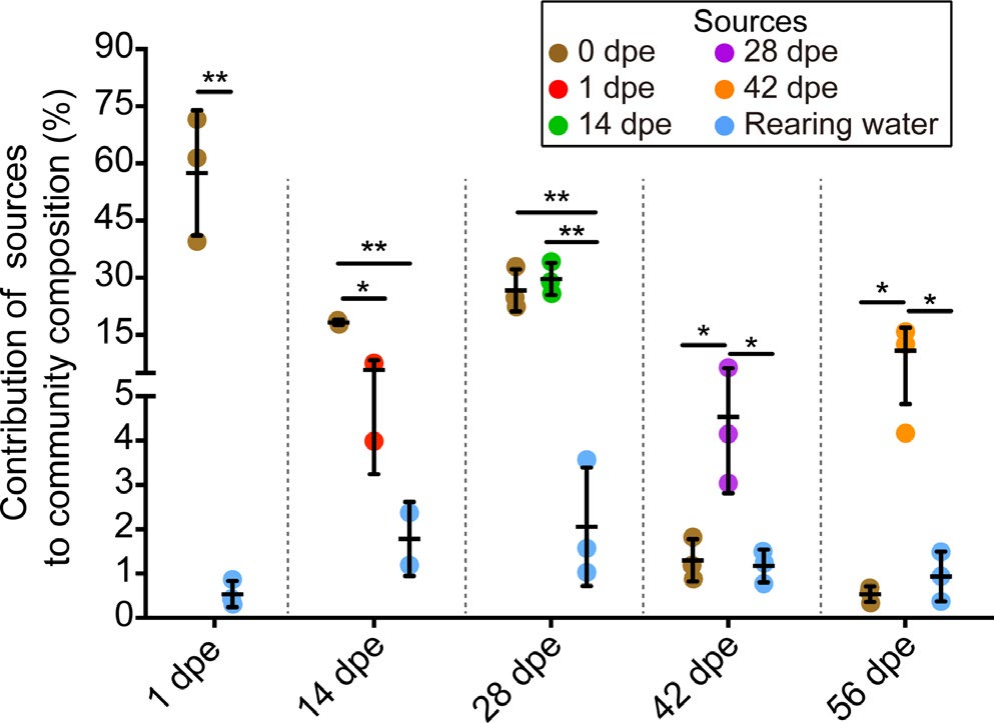
SourceTracker analysis showing the contributions of different source communities to the bacterial communities at different stages of gut regeneration. One asterisk (*) indicates statistical significance (*p* < 0.05), and two asterisks (**) indicate extreme statistical significance (*p* < 0.01)

The potential sources of the gut bacterial communities observed during gut regeneration were predicted by source tracking (Figure 6). Generally, the bacterial communities at 1–28 dpe were primarily sourced from the gut communities at 0 dpe and the preceding sampling time. However, the proportion of ASVs sourced from the gut at 0 dpe was significantly decreased at 42 and 56 dpe. The bacterial communities at 42 and 56 dpe were mainly sourced from those at 28 and 42 dpe, respectively. In addition, only a small proportion of the *A*. *japonicus* gut bacterial community observed during gut regeneration originated from the rearing water, with observed proportions ranging from 0.43% ± 0.07% to 2.77% ± 2.59%.

### 3.6 Ecological processes involved in the reconstruction of the gut bacterial community

**FIGURE 7:**
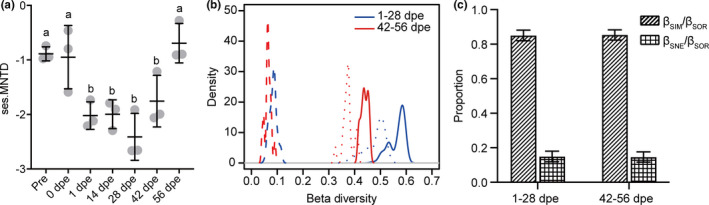
Analysis of the ecological processes of the bacterial community at different *A*. *japonicus* gut regeneration stages. (a) Changes in ses.MNTD over the gut regeneration period. Different lowercase letters above the error bars indicate significant differences among groups. (b) The partitioning of β_SOR_ (solid line) into β_SIM_ (dotted line) and β_SNE_ (dashed line). (c) The proportions of β_SIM_ and β_SNE_ in β_SOR_. Lowercase letters above the error bars indicate significant differences in β_SIM_/β_SOR_ among groups and capital letters indicate significant differences in β_SNE_/β_SOR_ among groups

The ses.MNTD values and the contributions of temporal turnover (β_SIM_) and nestedness (β_SNE_) to beta diversity in the different gut regeneration stages were calculated to infer the processes of bacterial community assembly. The ses.MNTD values were all less than zero (Figure 7‐a), suggesting that the bacterial communities tended to be more phylogenetically clustered than expected by chance. The absolute value of ses.MNTD significantly increased after evisceration, peaking at 28 dpe and then decreasing, reaching the pre‐evisceration level at 56 dpe. Beta diversity partitioning was performed based on the three clusters identified in the beta diversity analysis. The distributions of β_SIM_ and β_SNE_ were similar among the different gut regeneration stages (Figure 7‐b), and the β_SIM_ value was much higher than the β_SNE_ value at each regeneration stage (Figure 7‐c). These results suggested that the reconstruction of the *A*. *japonicus* gut bacterial community is primarily governed by turnover.

## 4 DISCUSSION

Sea cucumber gut regeneration is a useful model system for studying organ regeneration and development (Candelaria et al., 2006; García‐Arrarás et al., 2018) as well as gut microbiota assembly. However, gut microbiota succession during gut regeneration in the sea cucumber and its ecological mechanisms remain poorly understood. In the present study, the succession of the gut bacterial community, host‐microbiota and bacterial‐bacterial interactions, and potential sources of the bacterial community were analyzed at different gut regeneration stages in *A*. *japonicus*.

The assembly of the gut microbial community is an important process in gut regeneration. In the present study, the diversity and composition of the *A*. *japonicus* gut bacterial community varied significantly throughout gut regeneration. Based on the beta diversity analysis of the bacterial community, the reconstruction of the gut bacterial community was divided into two distinct stages, an initial stage (1–28 dpe) and a later stage (42–56 dpe), which correspond to the lumen formation stage and the gut differentiation and growth stage, respectively (Zhang et al., 2017). Moreover, the microbial community composition in the sea cucumber gut at 42–56 dpe was notably different from that at the initial stage, suggesting the formation of a novel microbiota during gut regeneration. Moreover, this result is consistent with previous findings in the sea cucumber *Sclerodactyla briareus* and *A*. *japonicus* (Weigel, 2020; Yamazaki et al., 2020). Similarly, the source tracking results suggest that the *A*. *japonicus* gut bacterial community is reconstructed from the bacterial community present in the residual esophagus and stomach after evisceration. This process may be an energy‐saving strategy by the host, increasing the host's ability to reduce energy consumption in its interactions with pathogens or foreign microorganisms in rearing water by hosting a largely stable bacterial community that can resist colonization by invaders (De Schryver & Vadstein, 2014). Therefore, the host can allocate as much energy as possible to the formation and development of the regenerated viscera. After the intestine lumen formed, the proportions of bacteria from 0 dpe notably decreased, and the bacteria from the preceding sampling time point significantly contributed to the bacterial population. This shift suggests that the bacteria in the initial stage were gradually replaced by other taxa, as indicated by the increase in competitive interactions at the later stage. Richness and evenness are considered important quantitative indices of microbial diversity (Purvis & Hector, 2000). In the present study, the Pielou evenness index was high at the initial stage. Previous studies have indicated that higher evenness in a microbial community may indicate a lower risk for pathogen invasion and functionality loss in natural ecosystems (Balvanera et al., 2005; De Schryver & Vadstein, 2014). Therefore, the higher evenness of the bacterial community observed in the present study may reduce the risk of pathogen invasion in the initial stage of gut regeneration in sea cucumbers.

Gut microbiota succession occurs due to host‐microbe and microbe‐microbe interactions, which are strongly influenced by host physiology and environmental factors (Stephens et al., 2016). Several studies have focused on the gut microbiota assembly that occurs during gut regeneration in sea cucumbers (Weigel, 2020; Yamazaki et al., 2020; Zhang et al., 2020). However, the samples collected in these studies were mainly of newly regenerated gut or feces after the lumen has formed, and little is known about the microbiota in the initial stage of regeneration. In the present study, the gut bacterial community in both the initial and later stages was studied to reveal the ecological processes of microbiota reconstruction in the sea cucumber. The ses.MNTD value can be used to determine the ecological processes that govern a community in terms of phylogenetic structure. In the present study, the absolute value of ses.MNTD peaked at 28 dpe, which indicated a stronger effect of deterministic processes (host selection) (Wang et al., 2013) in the initial stage than in the later stage. This result was consistent with the results of the immunoenzyme assay. Thus, ACP, AKP, SOD, and LZM activities have been monitored in previous studies to evaluate immune status during gut regeneration (Wang et al., 2008; Wang et al., 2008; Zang et al., 2012). In the present study, the RDA demonstrated that among the studied enzymes, ACP and SOD had the greatest effects on the structure of the bacterial community, especially in the initial stage. Previous studies have shown that many immune‐related genes are differentially expressed between the post‐regeneration stage and the initial stage of gut regeneration (Ramírez‐Gómez et al., 2008; Zhang et al., 2017), demonstrating that the host immune status may contribute to the assembly of the bacterial community in the initial stage. Source tracking analysis revealed that a few of the gut bacteria present in *A*. *japonicus* during regeneration originated from the rearing water, which is consistent with findings in *S*. *briareus* (Weigel, 2020). These results suggest that the bacteria from the surrounding environment that enter the gut undergo strong host selection. Taken together, our findings suggest that the host has a greater influence on bacterial community structure in the initial stage of *A*. *japonicus* gut reconstruction than in the later stage.

The strong deterministic process in the initial stage may lead to the selection of specific bacterial taxa by the host. Many members of *Vibrio* are pathogens of sea cucumbers. In this study, the abundance of *Vibrio* was significantly decreased in the initial stage; a reduced abundance of *Vibrio* during this stage might reduce the risk of disease onset. *Roseburia* (class *Clostridia*), *Prevotella*, and *Muribaculaceae* (class Bacteroidia), which were abundant in the normal gut, maintained their high abundance in the initial stage of regeneration. *Clostridia* members, including *Roseburia*, have been demonstrated to play crucial roles in the maintenance of gut homeostasis, with functions including resisting pathogen infection and gut inflammation (Kasahara et al., 2018; Kim et al., 2017; Lopetuso et al., 2013). As the expression of inflammatory response‐related genes has been reported to be upregulated in the initial stage (Rojas‐Cartagena et al., 2007), the prevalent *Clostridia* members probably contribute to reducing excessive inflammatory responses during this stage. Moreover, genes associated with carbohydrate utilization are enriched in the rapidly regenerating gut of *A*. *japonicus* (Zhang et al., 2020). *Prevotella* and *Muribaculaceae* are characterized by high utilization rates of diverse complex carbohydrates (Accetto & Avguštin, 2015; Lagkouvardos et al., 2019), suggesting that their high abundance benefits lumen formation in the initial stage.

Gut bacteria establish complex networks by interacting with each other and perform system functions through flows of energy, matter, and information. In the present study, the bacterial networks were simple, and the percentage of positive co‐occurrences was highest in the initial stage, possibly due to the host selection of synergetic bacterial species that are beneficial for gut regeneration. As the influence of the host weakens in the later stage of gut regeneration, the bacterial network becomes more complex, and the proportion of negative co‐occurrences increases, indicating that more competitive interactions are occurring in these networks. Competitive interactions have been shown to increase the stability of gut microbial communities (Coyte et al., 2015), indicating that the bacterial community was more stable in the later stage. These findings indicate that bacterial interactions vary significantly during the gut regeneration process, and the variations may facilitate the regeneration and functional recovery of the *A*. *japonicus* gut.

## 5 CONCLUSION

**FIGURE 8:**
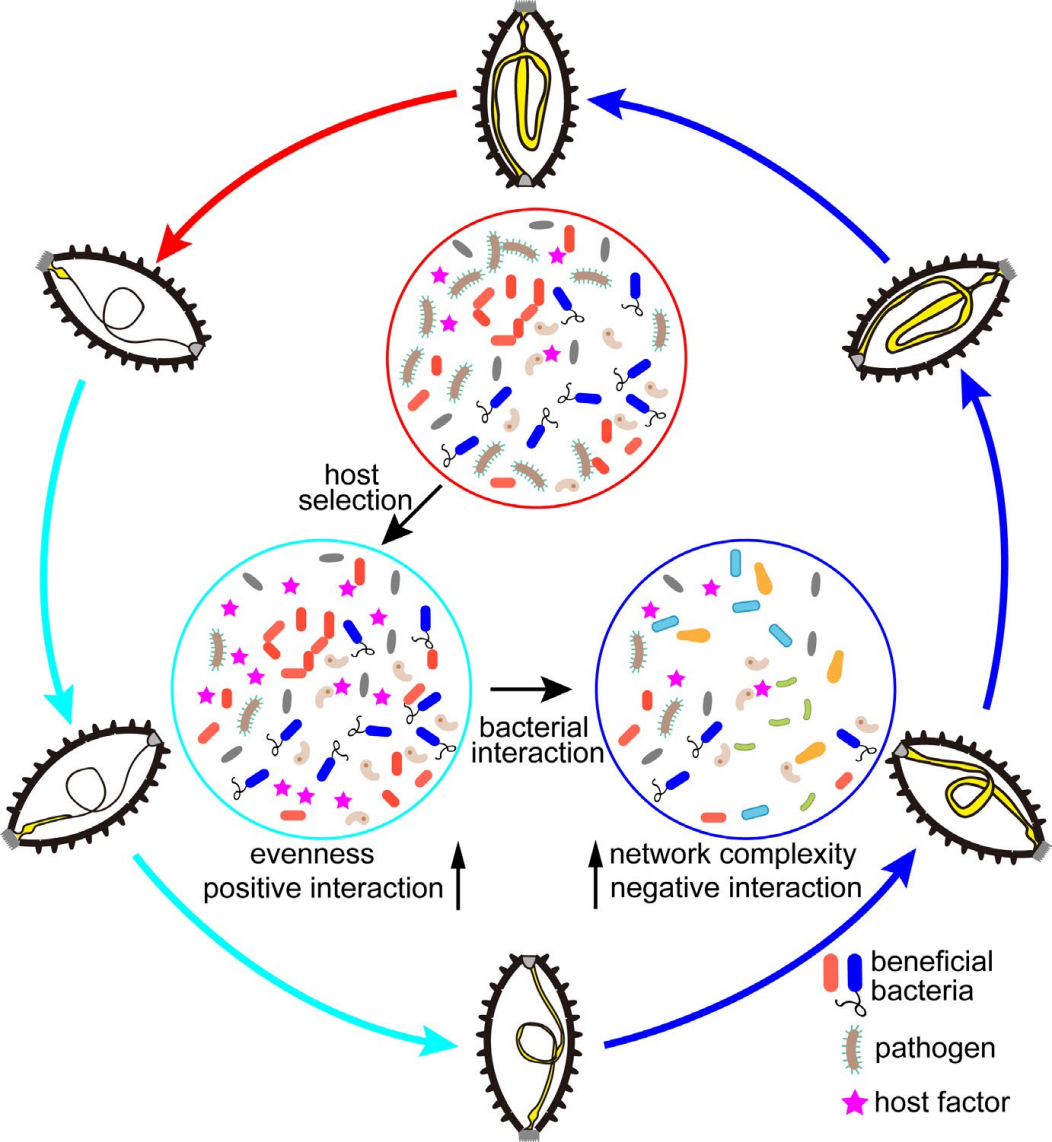
Schematic summary of the bacterial community dynamics and their controlling factors during bacterial community reconstruction. The red arrow indicates the evisceration of the *A*. *japonicus* gut. The red circle highlights the bacterial community in the residual digestive tract. The cyan arrow indicates the lumen formation process in the regenerating gut. The cyan circle highlights the bacterial community in the initial stage of reconstruction. The blue arrow indicates the differentiation and growth processes in the regenerating gut. The blue circle highlights the bacterial community in the later stage of reconstruction

In summary, reconstruction of the gut bacterial community of *A*. *japonicus* is initiated from the species pool in the residual esophagus and stomach after evisceration and involves two distinct and dynamic stages (Figure 8). In the initial stage, host factors are the primary drivers of bacterial community assembly, and host selection inhibits pathogenic taxa and maintains the population of beneficial taxa (Figure 8). In the later stage, the interactions among bacteria are more complex and competitive, making the community more stable. Moreover, these bacterial community dynamics are likely to provide the host with energetic and immune benefits that promote the regeneration and functional recovery of the *A*. *japonicus* gut.

## CONFLICT OF INTEREST

None declared.

## AUTHOR CONTRIBUTIONS

**Zichao Yu:** Conceptualization (lead); Formal analysis (lead); Funding acquisition (lead); Investigation (lead); Resources (supporting); Supervision (equal); Validation (equal); Visualization (lead); Writing‐original draft (equal); Writing‐review & editing (equal). **Zhuang Xue:** Investigation (equal); Resources (lead); Writing‐original draft (supporting); Writing‐review & editing (supporting). **Chao Liu:** Investigation (equal); Writing‐original draft (supporting); Writing‐review & editing (supporting). **Anguo Zhang:** Investigation (supporting); Resources (supporting); Writing‐original draft (supporting); Writing‐review & editing (supporting). **Qiang Fu:** Formal analysis (supporting); Investigation (supporting); Visualization (supporting); Writing‐original draft (supporting); Writing‐review & editing (supporting). **Kun Yang:** Investigation (supporting); Visualization (supporting); Writing‐original draft (supporting); Writing‐review & editing (supporting). **Fang Zhang:** Investigation (supporting); Visualization (supporting); Writing‐original draft (supporting); Writing‐review & editing (supporting). **Liyuan Ran:** Conceptualization (lead); Formal analysis (supporting); Investigation (supporting); Supervision (lead); Validation (lead); Writing‐original draft (equal); Writing‐review & editing (lead).

## ETHICS STATEMENT

None required.

## Data Availability

All data are provided in full in this paper except for the raw sequence data which are available at https://www.ncbi.nlm.nih.gov/bioproject/PRJNA720500.

## APPENDIX 1

**TABLE A1:** One‐way Analysis of Similarity (ANOSIM) based on Bray‐Curtis distance for bacterial communities in different stages of gut regeneration

	cluster 1	cluster 2	cluster 3
cluster 1		0.89 *p* = 0.0018	0.91 *p* = 0.0069
cluster 2	0.89 *p* = 0.0018		0.75 *p* = 0.0006

Note

Global R = 0.799, *p* = 0.0001, 9999 permutations.

**TABLE A2:** Effect of immunoenzymes on bacterial community structure based on RDA analysis

Name	Explains (%)	*p*
ACP	23.9	0.002
SOD	23.4	0.002
AKP	9.2	0.058
LYZ	7.4	0.112

**TABLE A3:** Properties of the networks of the bacterial communities in different gut regeneration stages

Properties	1–28 dpe	28–56 dpe
Nodes	288	193
Edges	2234	1628
Average degree (avgK)^a^	15.51	16.87
Average path distance (GD)^b^	4.09	3.07
R^2^ of power law	0.53	0.44
Positive edges (%)	88.09	28.19
Modules	11	12
Modularity	0.26	0.18

^a^ A higher avgK value indicates a more complex network.

^b^ A lower GD value indicates the nodes in the network are closer.
